# Association of the *eNOS* Gene Intron 4 VNTR Polymorphism with Susceptibility to Preeclampsia and Its Severity in an Algerian Cohort

**DOI:** 10.3390/genes17040398

**Published:** 2026-03-30

**Authors:** Sara Mimi Atmani, Faiza Bouldjennet, Yasmine Khalili, Nabyla Feghoul, Amel Dammene Debbih

**Affiliations:** 1 Laboratory of Cellular and Molecular Biology, Faculty of Biological Sciences, University of Sciences and Technology Houari Boumediene, PB 32, El Alia, Bab Ezzouar, Algiers 16111, Algeria; faiza.bouldjennet_fsb@usthb.edu.dz; 2 Research Laboratory, Biodiversity, Biotechnology, Environment and Sustainable Development, Department of Biology, Faculty of Sciences, M’Hamed Bougara University, Boumerdes 35000, Algeria; 3 Department of Obstetrics and Gynaecology, Ibn-Ziri Hospital, EPH Bologhine, Algiers 16000, Algeria; khalili.yasmine99@gmail.com (Y.K.); a.dammenedebbih@univ-alger.dz (A.D.D.); 4 Department of Obstetrics and Gynaecology, El-Tayeb Bougasmi Hospital, EPH Zeralda, Algiers 16000, Algeria; nabyla.feghoul@gmail.com; 5 Faculty of Medicine, University of Algiers 1, Algiers 16000, Algeria

**Keywords:** preeclampsia, *eNOS*, intron 4 VNTR, −786 T>C variant, PCR-RFLP

## Abstract

Background/Objectives: Preeclampsia (PE) is the primary cause of maternal and perinatal morbidity and mortality on a global scale. It is driven by a multifactorial aetiology, in which genetic factors involved in blood pressure regulation, including the endothelial nitric oxide synthase (*eNOS*) gene, play an important role. This study aimed to investigate the association between *eNOS* gene polymorphisms and the development and severity of PE in an Algerian cohort. Methods: A total of 305 Algerian women, comprising 124 patients with PE and 181 healthy controls, were genotyped for two polymorphisms: intron 4 variable number tandem repeat (VNTR) (4a/4b) and the −786 T>C promoter variant. Genotyping was performed using standard polymerase chain reaction (PCR) for VNTR and polymerase chain reaction–restriction fragment length polymorphism (PCR-RFLP) for the −786 T>C variant. Results: Our results revealed no significant difference in the allelic or genotypic frequencies of the −786 T>C polymorphism between the cases and controls (*p* > 0.05). Interestingly, the frequency of the protective “4b” allele was significantly lower in cases than in controls (odds ratio (OR) = 0.400 [0.278–0.575]; *p* < 0.0001). However, the “4a” allele and the 4a/4a genotype were significantly associated with an increased risk of preeclampsia (OR = 2.50 [1.74–3.59], *p* < 0.0001; and OR = 6.20 [2.85-13.50], *p* < 0.0001, respectively). Furthermore, they were correlated with disease severity (allelic: OR = 2.76 [1.60–4.75], *p* = 0.0002; and genotypic: OR = 4.64 [1.83-11.78], *p* = 0.0003). Conclusions: These findings support the potential role of the *eNOS* VNTR 4a/4b polymorphism in both the risk and severity of preeclampsia in the Algerian population.

## 1. Introduction

Hypertensive disorders during pregnancy constitute a major contributor to maternal and perinatal morbidity and mortality worldwide [1]. Among these conditions, preeclampsia (PE), defined as the new onset of hypertension accompanied by proteinuria after 20 weeks of gestation in previously normotensive women, is a major clinical concern [2]. With an estimated global prevalence of 2–8% of pregnancies, PE is associated with substantial adverse outcomes for both the mother and foetus, including an elevated risk of preterm delivery, foetal growth restriction, and long-term cardiovascular complications [2,3].

Although the aetiology of PE remains largely unresolved, the two-stage model proposed in 1991 remains a widely accepted framework for understanding the pathogenesis of this multifaceted disease [4,5]. As previously mentioned, the initial stage of preeclampsia is characterised by abnormal placentation resulting from insufficient trophoblastic invasion of the maternal endometrium. This precedes the onset of clinical manifestations. Inadequate placentation, likely due to alterations in metabolic pathways involved in nitric oxide production, renders the placenta hypoxic or ischaemic, leading to the release of anti-angiogenic and inflammatory factors into maternal circulation [6]. These soluble factors induce systemic endothelial dysfunction, which leads to clinical features of PE (stage two), such as hypertension and proteinuria [5,7].

Certain genes have been shown to play key roles in PE pathophysiology [8,9]. One such gene encodes endothelial nitric oxide synthase (eNOS, also known as NOS3), a constitutively expressed enzyme responsible for nitric oxide (NO) production in endothelial cells and for blood pressure regulation. In the placenta, NO modulates vascular tone and blood flow, with its production increasing throughout pregnancy. Dysregulation of this pathway may contribute to the endothelial dysfunction observed in PE [5,10].

The *eNOS* gene is located on chromosome 7q35–36 and comprises 26 exons separated by 25 introns. It encodes a 135 kDa protein comprising 1203 amino acids [11,12]. The *eNOS* gene displays substantial genetic variability, and several studies have reported that certain genetic variants are associated with diminished eNOS expression and activity, which consequently results in decreased NO bioavailability [13,14]. Among these polymorphisms, the most extensively investigated are: (i) a single-nucleotide polymorphism (SNP) at position −786 in the promoter region (−786T>C; rs2070744), which has been shown to reduce promoter activity by approximately 50%; (ii) a coding SNP at position 894 (c.894G>T, p.Glu298Asp; rs1799983) in exon 7, potentially affecting NOS3 localisation to the caveolar membrane; and (iii) a 27 bp variable number of tandem repeats (VNTR) located in intron 4 (rs61722009), comprising the 4b allele (five repeats) and the 4a allele (four repeats), which has been reported to influence the production of a 27-nucleotide small RNA, thereby decreasing NOS3 mRNA and protein levels [15,16,17,18].

These genetic polymorphisms, which influence eNOS expression or activity, have been studied worldwide to assess their association with the risk of developing preeclampsia. However, studies on the genetic predisposition to preeclampsia are limited in our geographical area. In this context, our study aimed to assess the relationship between *eNOS* −786T>C (rs2070744) and intron 4 VNTR (rs61722009) variants and the risk of developing preeclampsia in pregnant women from Algeria.

## 2. Materials and Methods

### 2.1. Study Participants

This case–control study included 305 unrelated Algerian women, of whom 124 had a confirmed diagnosis of preeclamptic pregnancy (case group) and 181 had healthy normotensive pregnancies (control group). Samples were recruited from the Obstetrics and Gynaecology departments (hospitalised and outpatient) of IBN-ZIRI Hospital, BACHIR MENTOURI Hospital, and NAFFISSA HAMOUD University Hospital in north-central Algeria.

Preeclampsia was diagnosed according to the International Society for the Study of Hypertension in Pregnancy (ISSHP) and the American College of Obstetrics and Gynaecology (ACOG), which define preeclampsia as gestational hypertension (systolic blood pressure ≥ 140 mmHg and/or diastolic blood pressure ≥ 90 mmHg on at least two measurements separated by a minimum interval of 6 h) occurring after 20 weeks of pregnancy, in association with one or more new-onset conditions, including proteinuria (≥0.3 g protein in a 24 h urine collection or ≥ +1 on a urine dipstick), any maternal organ dysfunction, or uteroplacental dysfunction [2,19].

Preeclampsia that develops before 34 weeks of gestation is usually defined as early-onset preeclampsia, whereas the one that develops at or after 34 weeks of gestation is defined as late-onset preeclampsia [20]. PE was considered severe if one of the following signs was present: severe hypertension (≥160/110 mmHg), proteinuria ≥ 2 g/24 h or > +2 dipstick, oliguria, persistent headache, visual disturbances, upper abdominal pain, convulsion, elevated serum creatinine, thrombocytopenia (platelet count < 100 ×10^9^/L), impaired liver function (marked elevation of serum transaminases), foetal growth restriction, and/or pulmonary oedema [2].

The case group, whose mean age was 33.31 ± 5.95 years (range: 19–45 years), included patients with or without complications of PE, such as eclampsia or HELLP syndrome, whereas the control group, whose mean age was 33.27 ± 6.19 years (range: 20–45 years), included normotensive women with normal pregnancy outcomes and without any symptoms of preeclampsia or its associated conditions. Subjects with multifetal pregnancies or pre-existing chronic hypertension, regardless of whether they had superimposed PE, were excluded from the study.

The study was approved by the Ethics Committee of Bologhine IBN ZIRI Hospital (reference number: CEB/0025/05), and written informed consent was obtained from all participants prior to their inclusion in the study.

### 2.2. Genetic Analysis

#### 2.2.1. DNA Extraction

Genomic DNA was extracted from peripheral blood samples collected in ethylenediaminetetraacetic acid (EDTA) tubes using the salting-out method [21]. The obtained DNA was subsequently dissolved in Tris-EDTA (TE) buffer (Sigma-Aldrich, St. Louis, MO, USA), and its quality and quantity were assessed by measuring the 260/280 wavelength ratio using a NanoDropTM One spectrophotometer (Thermo Fisher Scientific Inc. Waltham, MA, USA).

#### 2.2.2. *eNOS* Variant Genotyping

Genotyping of intron 4 VNTR and −786T>C polymorphisms of the *eNOS* gene was carried out using polymerase chain reaction (PCR) and polymerase chain reaction–restriction fragment length polymorphism (PCR-RFLP) techniques, respectively. Details regarding the primer sequences, amplification conditions, expected amplicon sizes, and restriction fragments are provided in Table 1. Both PCR amplifications were performed using a MiniAmp™ Thermal Cycler (Thermo Fisher Scientific Inc., Waltham, MA, USA) in 25 µL of the reaction mixture containing: 200 ng of genomic DNA, 0.5 μM of each primer, 0.312 U of Taq DNA polymerase, 200 μM of each dNTP (dATP, dGTP, dCTP, dTTP), 1.5 mM of MgCl2, and 1X enzyme buffer (Promega, Madison, WI, USA) with nuclease-free water added to volume.

Amplification of the region containing the −786T>C polymorphism was followed by enzymatic digestion of 10 µL of the PCR product using 1 µL of MspI Enzyme (10 U/µL; Thermo Fisher Scientific Inc., Waltham, MA, USA; Cat. No. ER0541) at 37 °C overnight, according to the manufacturer’s instructions.

**Figure 1:**
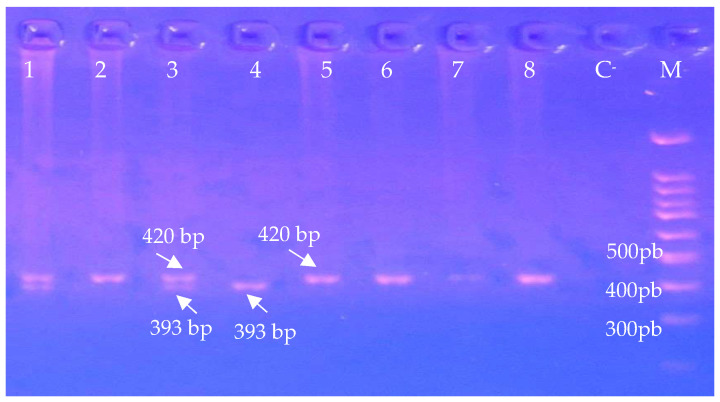
Representative electrophoretic profile of intron 4 polymorphism of *eNOS* gene. Lanes 1 and 3: 4a/4b genotype; Lanes 2, 7 and 8: 4b/4b genotype; Lane 4: 4a/4a genotype; Lane C^-^: negative control; Lane M: 100 bp DNA Ladder.

The PCR products and fragments obtained by restriction were visualised under UV light after 2.5% agarose gel electrophoresis stained with ethidium bromide solution. A representative electrophoretic profile of the intron 4 polymorphism of the *eNOS* gene is shown in Figure 1.

All samples were coded and genotyped without separating the cases and controls. To ensure genotyping accuracy, several quality control measures were implemented: negative controls without DNA were included in each PCR run to monitor potential contamination; all samples lacking an initial genotype call were rerun; and approximately 10% of the samples were randomly selected for repeat genotyping, which showed 100% concordance.

### 2.3. Statistical Analysis

Clinical and biological characteristics (age, blood pressure, body mass index (BMI), gestational age at delivery, proteinuria, and birth weight) of the patients and controls are expressed as mean ± standard deviation (SD) or median [Q1; Q3], and comparisons were conducted using the Mann–Whitney test with PAST software (version 4.0, Natural History Museum, University of Oslo, Oslo, Norway).

For each SNP studied, the genotype distribution was evaluated for deviation from the Hardy–Weinberg equilibrium using a Chi-square test within the control cohort. Differences in genotype and genotypic combination frequencies were determined using the Chi-square or Fisher’s exact test via SNPStats program (available at https://www.snpstats.net/start.htm accessed on 30 December 2025). Multivariable logistic regression analyses were then performed to evaluate the association between *eNOS* polymorphisms and preeclampsia risk, adjusting for maternal age, BMI, and parity as potential confounders. For the genotypic, dominant, and recessive models, analyses were conducted using SNPStats, while the allelic model was analysed using R statistical software (version 4.2.2, R Foundation for Statistical Computing, Vienna, Austria). Regression coefficients (β), adjusted odds ratios (ORs), and 95% confidence intervals (CIs) were estimated, with significance assessed by the Wald test. While the nominal significance level was set at *p* < 0.05, a Bonferroni-corrected threshold of *p* < 0.0125 was applied to account for the four genetic models tested.

## 3. Results

### 3.1. Clinical Characteristics According to Preeclampsia Status

This study included 124 women diagnosed with preeclampsia (PE) and 181 normotensive pregnant controls, all of whom were recruited from the north-central region of Algeria. Table 2 outlines the clinical characteristics of the cases and controls. No statistically significant difference was observed in the mean age between the control and preeclampsia groups. Yet, the PE group exhibited notably higher systolic and diastolic blood pressures compared to healthy pregnant women. In addition, there was a significant association between PE and overweight status (BMI ≥ 25 kg/m^2^), parity, and gravidity.

Pregnant women with PE delivered significantly earlier than normotensive controls (median [Q1; Q3]: 35 [34; 37] vs. 39 [38; 39] weeks). Furthermore, women with PE were more susceptible to pregnancy loss than those in the control group. Owing to the well-known maternal and foetal complications associated with PE and the urgency of performing a caesarean section in such cases, the caesarean delivery rate in our case study was significantly higher.

### 3.2. Clinical Characteristics According to Preeclampsia Severity

Based on the American College of Obstetricians and Gynaecologists criteria for defining preeclampsia severity [2], the preeclamptic patients were subdivided into 82 mild-PE and 42 severe-PE groups. Clinical data analysis showed that severe features were significantly associated with early-onset preeclampsia (before 34 weeks of pregnancy) (Table 2). Maternal age did not differ significantly between the two subgroups. However, systolic and diastolic blood pressures, as well as BMI, were significantly higher in the severe preeclampsia group. Furthermore, gestational age at delivery was notably lower in the group with severe preeclampsia, which partly explains the significantly lower foetal birth weight in this group (Table 2).

### 3.3. eNOS Genotype Distribution in the Studied Groups

The genotype distribution of *eNOS* −786T>C (rs2070744) and intron 4 VNTR 4a/4b (rs61722009) variants was found to be in Hardy–Weinberg equilibrium in our study population (*p* = 0.25 and *p* = 0.33, respectively). The genotypic and allelic distributions of the rs2070744 polymorphism are detailed in Table 3, where no significant association was observed between the T and C alleles (*p* > 0.05). Similarly, no association was detected between this polymorphism and PE severity in the present study (*p* > 0.05).

In contrast, the analysis of intron 4 VNTR (rs61722009) revealed significant differences between the groups (Table 4). The occurrence of the “4b” allele and the 4b/4b genotype was markedly lower in individuals with PE compared to the control group (61 vs. 80% and 47.6 vs. 65.2%, respectively), suggesting a potential protective effect (OR (95% CI) = 0.400 (0.278–0.575)) for the “4b” allele. Moreover, the “4a” allele and the 4a/4a genotype were observed to be significantly more prevalent among PE cases than in controls, indicating a possible role in increasing susceptibility to preeclampsia development (39 vs. 20% and 25 vs. 5.5%, OR (95% CI) = 2.50 (1.74–3.59) and OR (95% CI) = 6.20 (2.85–13.50)), respectively). In multivariable logistic regression analyses, even after adjusting for maternal age, BMI, and primigravidity, the 4a/4a genotype continued to show a significant association with an elevated risk of preeclampsia (adjusted OR (95% CI) = 6.66 (2.92–15.20)) (Table 4). The statistical significance of these associations persisted following the application of a Bonferroni correction for multiple comparisons (k = 4), with adjusted *p*-values of 0.0004. Additionally, post hoc power analysis revealed exceptionally high statistical power for the case–control analysis (> 99% for both recessive and allelic models).

When comparing mild and severe preeclampsia, the genotype frequencies of 4b/4b, 4a/4b, and 4a/4a were 53.7%, 31.7%, and 14.6% in mild PE, and 35.7%, 19.1%, and 45.2% in severe PE, respectively. Carriage of the “4a” allele was significantly more prevalent in cases with severe preeclampsia (OR (95% CI) = 2.76 (1.60–4.75)) (Table 5), suggesting a possible association with disease severity. Logistic regression analysis further confirmed that the 4a/4a genotype was significantly associated with severe preeclampsia (adjusted OR (95% CI) = 4.60 (1.78–11.92)) (Table 5). After Bonferroni correction, the associations remained statistically significant (*p* = 0.0008), and post hoc power analysis confirmed a statistical power of 84.6% for detecting the association between the 4a/4a genotype and severe preeclampsia.

The corresponding regression coefficients and additional statistical analyses, including power analysis and Bonferroni-corrected results, are provided in Supplementary Tables S1–S4.

## 4. Discussion

As previously outlined, abnormal placentation is the initial event in the pathogenesis of preeclampsia. A reduction in nitric oxide (NO) bioavailability has been recognised as a key contributor to impaired maternal–placental perfusion, thereby promoting this defective placental development [24,25,26]. Several factors are known to alter NO signalling and are associated with an increased risk of preeclampsia [27], among which alterations in *eNOS* regulation or function, mainly due to *eNOS* gene polymorphisms such as the −786T>C polymorphism in the promoter region, the 4a/4b polymorphism in intron 4, and the Glu298Asp polymorphism in exon 7, have been implicated [5,28].

To our knowledge, this is the first study to investigate the association between *eNOS* gene polymorphisms (rs2070744 and rs61722009) and the risk and severity of preeclampsia in a cohort of Algerian women.

The involvement of BMI, maternal age, and parity as maternal risk factors for the onset of preeclampsia is well established [2,29]. In fact, previous studies have reported that women with increased age and BMI face a doubled risk of developing PE, and the risk increases by approximately 30% with each successive year of age [30,31]. Furthermore, two-thirds of preeclamptic cases are nulliparous, largely due to the absence of prior maternal immunological and vascular adaptations during pregnancy [29,32].

In the current study, there was no significant difference in maternal age between the study groups (controls vs. cases: *p* = 0.9278; mild-PE vs. severe-PE: *p* = 0.8631). However, the risk of preeclampsia and its severity were significantly associated with obesity (BMI ≥ 25 kg/m^2^ before pregnancy) and nulliparity. To assess the effect size of the studied gene variants in our case–control population and their involvement as independent risk factors for preeclampsia, the statistical parameters of these variants were adjusted for maternal age, BMI, and parity. The independent contribution of the investigated *eNOS* variants to preeclampsia susceptibility was assessed using multivariate logistic regression models adjusted for maternal age, BMI, and parity.

The −786C allele at the *eNOS* gene promoter reduces promoter activity, which affects *eNOS* expression and activity [33]. Genetic analysis of the −786T>C polymorphism in the *eNOS* gene showed no statistical differences in the allelic (*p* = 0.9694), dominant (*p* = 0.23), or recessive models (*p* = 0.13) between preeclamptic women and healthy controls. These results strongly suggest a lack of association between this variant and the risk of preeclampsia in our study population. Our findings align with those of other studies conducted among different ethnic groups, including Caucasian [34], Asian [28,35], African [8], and mixed populations [36], in which a non-significant association was observed between the CC genotype of the *eNOS* gene variant at the −786T>C position and the risk of preeclampsia. Moreover, Fondjo et al. demonstrated that the −786T>C variant did not significantly influence circulating NO levels in Ghanaian women with preeclampsia, further supporting the limited functional relevance of this variant in the pathogenesis of the disease [8].

In contrast to our observations, previous studies conducted in Serbian and Pakistani populations have reported a significant association between the *eNOS* −786T>C polymorphism and susceptibility to preeclampsia [18,37]. The inconsistency between these findings and ours may be due to variations in sample size and ethnicity between the studies. In this regard, a meta-analysis by Zeng et al. highlighted substantial interethnic variability in the distribution of the −786T>C polymorphism among women with preeclampsia, supporting the hypothesis that genetic effects may differ according to population-specific backgrounds [38].

Interestingly, statistical analysis in our cohort revealed that the TC genotype was significantly more prevalent among the control group than the case group (adjusted *p* = 0.036) and was associated with a 43% lower risk of developing preeclampsia in those who carried it (adjusted OR (95% CI) = 0.57 (0.34–0.97)). Although this finding suggests a potential protective effect of the TC genotype, it is important to interpret this result with caution. Further studies involving larger cohorts are required to confirm this preliminary observation.

The *eNOS* VNTR 4a/4b polymorphism has gained attention owing to its potential functional relevance in modulating eNOS activity. It is characterised by a variable number of tandem repeats, with the “4a” allele containing four repeats and the “4b” allele containing five repeats [39]. In the present study, carriage of the “4a” allele was significantly associated with an increased risk of preeclampsia, conferring approximately a sixfold higher susceptibility after adjustment for confounding factors (adjusted OR (95%CI) = 6.16 (2.76–13.74)). This finding suggests that the “4a” allele may contribute to genetic susceptibility to preeclampsia in the studied population. In contrast, the 4b/4b genotype was significantly more frequent among healthy controls than among preeclamptic women (adjusted *p* = 0.0035), supporting the possible protective role of this genotype against PE development. These observations are consistent with the findings of Sljivancanin Jakovljevic et al., who reported an approximately eightfold increased risk of preeclampsia among homozygous 4a/4a carriers compared with 4b/4b individuals [18]. Similarly, Groten et al. demonstrated that carriers of the “4b” allele exhibited a reduced risk of preeclampsia in cohorts of Caucasian and African women. Furthermore, Alpoim et al. showed that participants with the 4a/4a genotype had significantly lower circulating NO levels compared to those carrying the 4b/4b or 4a/4b genotypes, suggesting a functional impact of these polymorphism genotypes [40].

Previous studies have reported inconsistent findings concerning the association between the *eNOS* VNTR 4a/4b polymorphism and PE in different populations. A comprehensive meta-analysis by Tesfa et al., incorporating 19 studies conducted in Caucasian [18,41], African [42], Asian [35,37], and mixed populations [36], reported a non-significant association between the *eNOS* VNTR 4a/4b polymorphism and the risk of developing PE [39]. As for the study conducted on a Chinese population, it showed that the frequency of the “4a” allele was significantly lower in patients with PE than in control subjects, evoking a protective effect of this allele [43]. The discrepancies between these findings and ours may be due to differences in ethnicity, genetic background, and preeclampsia aetiology.

We also investigated the involvement of the *eNOS* VNTR 4a/4b polymorphism in PE severity. Statistical analysis revealed a significant association between the 4a/4a genotype and preeclampsia severity, suggesting that this genotype may be associated with preeclampsia severity in the Algerian population.

The strength of the current study is that, to the best of our knowledge, it is the first to assess the distribution of *eNOS* variants (rs2070744 and rs61722009) in the Algerian population, with the aim of identifying genetic factors associated with the risk of developing preeclampsia and its severity in this population. Its second strength is the quantification of the effect size of the gene variants under investigation, adjusted for recognised maternal risk factors for preeclampsia like maternal age, parity, and being overweight (BMI ≥ 25 kg/m^2^).

The primary limitation of our investigation is that the study population was restricted to a single geographical region of Algeria (northern Algeria), which may not fully represent the genetic diversity of the entire country. Future studies should include a larger and more geographically diverse cohort, particularly from southern Algeria, to enable comparative analyses and strengthen the generalisability of the findings.

## 5. Conclusions

In conclusion, our findings suggest a significant genetic association between the *eNOS* VNTR 4a/4b polymorphism and the risk and severity of preeclampsia in the Algerian population. These results support the potential involvement of *eNOS* in the pathogenesis of this complex disease. Further studies involving larger cohorts and complementary molecular approaches integrating multiple genetic polymorphisms are required to confirm and extend these findings to better identify the genetic factors that predispose individuals to develop preeclampsia.

## Figures and Tables

**Table 1:** PCR and PCR-RFLP conditions for *eNOS* variant genotyping.

*eNOS* Variant	Primers	Amplification Conditions	PCR Product Size (bp)	Restriction Fragment Length (bp)	Reference
g.−786T>C (rs2070744)	F: 5′-TGGAGAGTGCTGGTGTACCCCA-3′ R: 5′-GCCTCCACCCCCACCCTGTC-3′	-Initial denaturation: 94 °C for 5 min -35 cycles of: Denaturation: 94 °C for 30 s Annealing: 59 °C for 30 s Extension: 72 °C for 30 s -Final extension: 72 °C for 7 min	180	TT: 140, 40 TC: 140, 90, 50, 40 CC: 90, 50, 40	[22]
VNTR 4a/4b (rs61722009)	F: 5′-AGGCCCTATGGTAGTGCCTT-3′ R: 5′-TCTCTTAGTGCTGTGGTCAC-3′	-Initial denaturation: 94 °C for 5 min -35 cycles of: Denaturation: 94 °C for 30 s Annealing: 59 °C for 30 s Extension: 72 °C for 1 min -Final extension: 72 °C for 7 min	4b/4b: 420 4a/4b: 420, 393 4a/4a: 393	-	[23]

**Table 2:** Clinical characteristics of the studied groups.

Parameters	Control Group (*n* = 181)	PE Group (*n* = 124)	*p*-Value *	Mild PE (*n* = 82)	Severe PE (*n* = 42)	*p*-Value ^#^
Age (year) ^(a)^	33 [28; 38.0]	34 [28.5; 38]	0.9278	33 [28.0; 38.0]	34 [28.0; 39.0]	0.8631
Blood pressure (mmHg) ^(a)^						
SBP	110 [110; 120]	110 [150; 170]	<0.0001	150 [140; 160]	170 [160; 173]	<0.0001
DBP	70 [60; 80]	100 [90; 110]	<0.0001	90 [90; 100]	110 [110; 120]	<0.0001
BMI (kg/m^2^) ^(a)^	25.5 [23.2; 29.9]	29.8 [25.1; 34.4]	<0.0001	35.6 [33.1; 39.1]	41.2 [39; 44.1]	<0.0001
Parity (%) ^(b)^						
Nulliparous	52 (29%)	57 (46%)	0.0082	41 (50%)	16 (38.1%)	0.4086
Primiparous	51 (28%)	28 (23%)		18 (22%)	10 (23.8%)	
Multiparous	78 (43%)	39 (31%)		23 (28%)	16 (38.%)	
Gravidity (%) ^(b)^						
primigravida	49 (27%)	50 (40%)	0.152	34 (41.5%)	16 (38.1%)	0.7175
multigravida	132 (73%)	74 (60%)		48 (58.5%)	26 (61.9%)	
Gestational age at delivery (weeks) ^(a)^	39 [38; 39]	35 [34; 37]	<0.0001	36 [34; 38]	34 [32; 34]	<0.0001
Type of delivery ^(b)^						
Vaginal delivery	142 (78%)	22 (18%)	<0.0001	21 (26%)	1 (2%)	0.0009
Caesarean section	39 (22%)	102 (82%)		61 (74%)	41 (98%)	
Abortion ^(b)^						
Yes	38 (21%)	43 (35%)	0.0079	30 (37%)	13 (31%)	0.5328
No	143 (78%)	81 (65%)		29 (69%)	52 (63%)	
Onset of preeclampsia ^(b)^						
<34 weeks	-	-	-	48 (59%)	37 (88%)	0.0009
≥34 weeks	-	-	-	34 (49%)	5 (12%)	
Proteinuria (g/24 h) ^(a)^	-	-	-	0.699 [0.5; 0.975]	4 [3; 5.53]	<0.0001
Birth weight (g) ^(a)^	-	-	-	2750 [2500; 2880]	2450 [2300; 2613]	<0.0001

PE, preeclampsia; SBP, systolic blood pressure; DBP, diastolic blood pressure; BMI, body mass index; (a) data described by median[first quartile; third quartile]; (b) data described by number of cases and percentages; *p*-values obtained from Chi-square test, Mann–Whitney test or Fisher’s exact tests; * *p*-values from comparison between control and PE groups; **^#^** *p*-values from comparison between mild and severe PE groups; -, not applicable; BMI values before pregnancy.

**Table 3:** Allelic and genotypic distribution of *eNOS* −786T>C polymorphism among cases and controls.

*eNOS* g.−786T>C	Controls (*n* = 181)	Cases (*n* = 124)	*p*-Value	OR (95% CI)	*p*-Value *	OR (95% CI) *
	N (%)	N (%)				
Genotype						
T/T	78 (43.1%)	62 (50%)		Reference (1.00)		Reference (1.00)
T/C	77 (42.5%)	36 (29%)	0.0164	0.59 (0.35–0.99)	0.036	0.57 (0.34–0.97)
C/C	26 (14.4%)	26 (21%)	0.13	1.26 (0.66–2.38)	0.18	1.25 (0.64–2.46)
Dominant model						
T/T	118 (65.2%)	59 (47.6%)		Reference (1.00)		Reference (1.00)
T/C-C/C	63 (34.8%)	65 (52.4%)	0.23	0.76 (0.48–1.20)	0.33	0.78 (0.48–1.28)
Recessive model						
C/C	10 (5.5%)	31 (25%)	0.13	1.58 (0.87–2.88)	0.18	1.54 (0.82–2.92)
T/T-T/C	171 (94.5%)	93 (75%)		Reference (1.00)		Reference (1.00)
Allelic model						
T	233 (64%)	160 (65%)		Reference (1.00)		Reference (1.00)
C	129 (36%)	88 (35%)	0.9694	0.993 (0.71–1.39)	0.9729	1.01 (0.70–1.44)

* *p*-values and odds ratios adjusted for maternal age, BMI, and parity.

**Table 4:** Allelic and genotypic distribution of *eNOS* VNTR 4a/4b polymorphism among cases and controls.

*eNOS* VNTR 4a/4b	Controls (*n* = 181)	Cases (*n* = 124)	*p*-Value	OR (95% CI)	*p*-Value *	OR (95% CI) *
	N (%)	N (%)				
Genotype						
4b/4b	118 (65.2%)	59 (47.6%)		Reference (1.00)		Reference (1.00)
4a/4b	53 (29.3%)	34 (27.4%)	0.7235	0.91 (0.55–1.52)	0.65	0.88 (0.51–1.53)
4a/4a	10 (5.5%)	31 (25%)	<0.0001	6.20 (2.85–13.50)	<0.0001	6.66 (2.92–15.20)
Dominant model						
4b/4b	118 (65.2%)	59 (47.6%)	0.0022	Reference (1.00)		Reference (1.00)
4a/4b-4a/4a	63 (34.8%)	65 (52.4%)		2.06 (1.29–3.29)	0.0035	2.10 (1.27–3.47)
Recessive model						
4a/4a	10 (5.5%)	31 (25%)	<0.0001	5.70 (2.68–12.14)	<0.0001	6.16 (2.76–13.74)
4b/4b-4a/4b	171 (94.5%)	93 (75%)		Reference (1.00)		Reference (1.00)
Allelic model						
4b	289 (80%)	152 (61%)		Reference (1.00)		Reference (1.00)
4a	73 (20%)	96 (39%)	<0.0001	2.50 (1.74–3.59)	<0.0001	2.58 (1.76–3.83)

* *p*-values and odds ratios adjusted for maternal age, BMI, and parity.

**Table 5:** Association of *eNOS* VNTR 4a/4b with the severity of preeclampsia.

*eNOS* VNTR4a/4b	Moderate PE (*n* = 82)	Severe PE (*n* = 42)	*p*-Value	OR (95% CI)	*p*-Value *	OR (95% CI) *
	N (%)	N (%)				
Genotype						
4b/4b	44 (53.7%)	15 (35.7%)		Reference (1.00)		Reference (1.00)
4a/4b	26 (31.7%)	8 (19.1%)	0.13	0.51 (0.21–1.25)	0.074	0.78 (0.28–2.15)
4a/4a	12 (14.6%)	19 (45.2%)	0.0003	4.64 (1.83–11.78)	0.0002	4.60 (1.78–11.92)
Dominant Model						
4b/4b	44 (53.7%)	15 (35.7%)	0.057	Reference (1.00)	0.089	Reference (1.00)
4a/4b-4a/4a	38 (46.3%)	27 (64.3%)		2.08 (0.97–4.48)		1.95 (0.90–4.25)
Recessive Model						
4a/4a	12 (14.6%)	19 (45.2%)	0.0003	4.82 (2.03–11.42)	0.0002	5.02 (2.07–12.22)
4b/4b-4a/4b	70 (85.4%)	23 (54.8%)		Reference (1.00)		Reference (1.00)
Allelic model						
4b	114 (70%)	38 (45%)		Reference (1.00)		Reference (1.00)
4a	50 (30%)	46 (55%)	0.0002	2.76 (1.60–4.75)	0.0004	2.68 (1.55- 4.67)

* *p*-values and odds ratios adjusted for maternal age, BMI, and parity.

## Data Availability

The datasets generated and/or analysed during the current study are available from the corresponding author upon reasonable request.

## Supplementary Materials

The following supporting information can be downloaded at: https://www.mdpi.com/article/10.3390/genes17040398/s1, Table S1: Multivariable logistic regression analysis of the association between the *eNOS* VNTR 4a/4b polymorphism and risk of preeclampsia, Table S2: Multivariable logistic regression analysis of the association between the *eNOS* VNTR 4a/4b polymorphism and severity of preeclampsia, Table S3: Genetic model analysis, multiple testing correction, and statistical power for the association between the *eNOS* VNTR 4a/4b and risk of preeclampsia, Table S4: Genetic model analysis, multiple testing correction, and statistical power for the association between the *eNOS* VNTR 4a/4b and severity of preeclampsia.

## Abbreviations

The following abbreviations are used in this manuscript:

ACOG	American College of Obstetrics and Gynaecology
BMI	Body Mass Index
CI	Confidence Interval
SBP	Systolic Blood Pressure
DBP	Diastolic Blood Pressure
eNOS	endothelial Nitric Oxide Synthase
ISSHP	International Society for the Study of Hypertension in Pregnancy
NOS	Nitric Oxide Synthase
NO	Nitric Oxide
PE	Preeclampsia
SNP	Single-Nucleotide Polymorphism
TE	Tris-EDTA
VNTR	Variable Number of Tandem Repeats
OR	Odds Ratio
