# Supplementary material for: Association of the eNOS Gene Intron 4 VNTR Polymorphism with Susceptibility to Preeclampsia and Its Severity in an Algerian Cohort
Source: Genes (Basel). 2026 Mar 30;17(4):398. doi: 10.3390/genes17040398 (PMC13116626; doi:10.3390/genes17040398)
Supplement: Supplementary file 1 [file genes-17-00398-s001.zip › Supplementary Materials ATMANI et al.docx]

**Supplementary Materials**

Table S1. Multivariable logistic regression analysis of the association between the *eNOS* VNTR 4a/4b polymorphism and risk of preeclampsia.

| **Genotype** | **β coefficient** | **Standard**  **Error (SE)** | **OR*** | **95% CI** | ***p*-value*** |
| --- | --- | --- | --- | --- | --- |
| 4b/4b | Reference | – | 1.00 | – | – |
| 4a/4b | -0.13 | 0.28 | 0.88 | 0.51–1.53 | 0.65 |
| 4a/4a | 1.90 | 0.41 | 6.66 | 2.92–15.20 | <0.0001 |

*Adjusted for maternal age, BMI, and parity; -, not applicable.

Table S2. Multivariable logistic regression analysis of the association between the *eNOS* VNTR 4a/4b polymorphism and severity of preeclampsia.

| **Genotype** | **β**  **coefficient** | **Standard**  **Error (SE)** | **OR*** | **95% CI** | ***p*-value*** |
| --- | --- | --- | --- | --- | --- |
| 4b/4b | Reference | – | 1.00 | – | – |
| 4a/4b | -0.25 | 0.52 | 0.78 | 0.28–2.15 | 0.074 |
| 4a/4a | 1.53 | 0.45 | 4.60 | (1.78-11.92) | 0.0002 |

*Adjusted for maternal age, BMI, and parity; -, not applicable.

**Table S3. Genetic model analysis, multiple testing correction, and statistical power for the association between the *eNOS* VNTR 4a/4b and risk of preeclampsia.**

| Genetic model | *p*-value* | Bonferroni *p*-value^#^ | Power (1−β) |
| --- | --- | --- | --- |
| Genotypic model  4a/4a vs 4b/4b (reference) | 0.0001 | 0.0004 | >99% |
| Recessive model  4a/4a vs (4a/4b + 4b/4b) | 0.0001 | 0.0004 | >99% |
| Dominant model  (4a/4a + 4a/4b) vs 4b/4b | 0.0035 | 0.014 | 71.4% |
| Allelic model  4a vs 4b | 0.0001 | 0.0004 | >99% |

*Adjusted for maternal age, BMI, and parity; ^#^Bonferroni correction applied for multiple testing.

Table S4. Genetic model analysis, multiple testing correction, and statistical power for the association between the *eNOS* VNTR 4a/4b and severity of preeclampsia.

| Genetic model | *p-value** | Bonferroni *p-value*^#^ | Power (1−β) |
| --- | --- | --- | --- |
| Genotypic model  4a/4a vs 4b/4b (reference) | 0.0002 | 0.0008 | 84.6% |
| Recessive model  4a/4a vs (4a/4b + 4b/4b) | 0.0002 | 0.0008 | 89.4% |
| Dominant model  (4a/4a + 4a/4b) vs 4b/4b | 0.089 | 0.356 | 22.6% |
| Allelic model  4a vs 4b | 0.0004 | 0.0016 | 86.3% |

*Adjusted for maternal age, BMI, and parity; ^#^Bonferroni correction applied for multiple testing.
